# The Big Five Personality Traits of Medical Students Who Choose a Community-Oriented Medicine Career

**DOI:** 10.7759/cureus.88056

**Published:** 2025-07-16

**Authors:** Hirofumi Kanazawa, Tomonari Yoshizawa, Ikuo Shimizu

**Affiliations:** 1 Department of Anesthesiology, University of Minnesota, Minneapolis, USA; 2 Department of Psychiatry, Shinshu University School of Medicine, Nagano, JPN; 3 Department of Medical Education, Chiba University, Chiba, JPN

**Keywords:** big-five personality, choice of career, community medicine, medical school students, personal traits, primary care medicine, rural healthcare, rural practice intention, student selection

## Abstract

Background

Personality traits may play a role in the likelihood of success in training and careers. Identifying personality characteristics associated with a preference for community-oriented medicine, defined as medical practice that integrates clinical care with the public health needs of a local area, may help reduce mismatches in regional quota programs (RQPs) during medical school admissions. Few studies have examined the relationship between personality traits and medical students' intentions to engage in community health care. Moreover, no studies have examined this relationship under the RQPs, which account for a considerable proportion of medical school enrollment in Japan. The objective of this study was to examine the personality characteristics associated with medical students’ aspirations toward community-oriented medicine.

Methods

This study analyzed the personality tendencies of community medicine-oriented students. All medical students in a medical school in Japan (n=750), who were selected into the RQP, RQP without loan (RQPWL), or standard program, were asked to complete the validated psychometric questionnaire (the shortened Japanese version of the 'big five' personality traits) to investigate the five major personality traits of neuroticism, extraversion, openness, conscientiousness, and agreeableness. A multivariable logistic regression was performed to assess the association between personality traits and willingness to engage in community health care. We also assessed the association between personality traits and admissions programs.

Results

In total, 591 students completed the questionnaire. The RQPWL was associated with grade (odds ratio (OR) 0.77, 95% confidence interval (CI) 0.63-0.94, p=0.011) and gender (OR 2.94, 95% CI 1.48-5.96, p=0.002). The RQP was associated with gender (OR 1.76, 95% CI 1.06-2.92, p=0.028), extraversion (OR 0.91, 95% CI 0.85-0.98, p=0.012), and conscientiousness (OR 1.05, 95% CI 1.00-1.10, p=0.0496). There were differences in the willingness to engage in community health care concerning gender (OR 1.57, 95% CI 1.04-2.36, p=0.031), admission programs (OR 4.32, 95% CI 2.16-8.90, p<0.001 for the RQPWL; OR 20.69, 95% CI 10.65-44.38, p<0.001 for the RQP). Higher openness to experience was associated with increased willingness to engage in community health care (OR 1.06, 95% CI 1.01-1.11, p=0.027), whereas higher extraversion was inversely associated (OR 0.94, 95% CI 0.89-1.00, p=0.046). A significant gender-by-RQPWL interaction was detected; compared to the general admission program (GAP), women admitted through RQPWL showed markedly higher odds of willingness to engage in community health care (OR 17.66, 95% CI 3.83-81.5, p<0.001).

Conclusion

Female students tended to be willing to engage in community-oriented medicine, and gender diversity can improve medical staff recruitment in rural areas. Fundamental differences were observed between the RQP and community medicine-oriented groups, demonstrating that the RQP may not adequately select students who want to engage in community-oriented medicine. A solution can be found by adjusting admissions to accept applicants with lower extraversion scores and enhancing openness to experience and agreeableness by providing cooperative learning education in medical schools.

## Introduction

The shortage of health professionals working in medically underserved areas is a global problem [1,2]. The rural medical workforce shortage results from various causes, including inadequate workforce policies guiding the number of doctors in training, changing patterns of employment of doctors as new graduates seek better work-life balance, and so on. A comprehensive, community-based approach is therefore needed. Community-oriented primary care (COPC) is an approach that integrates the key components of primary health care and community medicine, developing them in a systematic, coordinated manner for practical application [3]. This concept supports community-oriented medicine, focusing on community needs, inclusive decision-making, and multidisciplinary cooperation. Rural healthcare is a practical approach to this concept. It applies people-centered principles in geographically isolated areas with severe constraints on human and medical resources. Even so, putting these ideas into practice in rural areas is still challenging. 

To cope with the shortage of health-care workers, several attempts have been made, including the introduction of financial incentives and employment limited to working in rural areas. Nevertheless, these measures do not rely heavily on easily measurable indicators, such as academic performance. To date, no definitive measures have been established. In particular, Japan is facing the most aged population in recorded world history; the elderly are more likely than younger people to have chronic diseases and complications, and hence are considered to have a higher demand for medical care [4]. In fact, demand for home healthcare is estimated to more than double by 2035 [5]. However, the number of physicians per 1,000 in Japan is 2.67 (as of 2020) [6], which is insufficient compared to the Organisation for Economic Co-operation and Development (OECD) average of 3.6 (as of 2019) [7]. Although the number of physicians is increasing, the maldistribution of the region has not improved [8]. Similarly, in the United States, where the number of physicians per 1,000 people is 2.77 (as of 2019) [6], the shortage of physicians in rural areas is well recognized, and it has been found that only 9% of physicians are responsible for medical care in rural areas where 20% of the population lives [7].

Medical schools at universities in Japan have established regional enrollment quotas to select students willing to work in regional healthcare in order to address shortages of physicians in specific regions or specialties. The regional quota system began spreading rapidly in Japan around 2006 [9]. A 2019 legal revision in Japan introduced two distinct quotas: the regional quota program (RQP) or chiiki-waku in Japanese, and the regional quota program without loan (RQPWL) or iimoto-waku in Japanese [10]. The RQP provides a non-repayable scholarship but requires graduates to serve in medically underpopulated areas for a set period after graduation, while RQPWL limits admission to applicants who have lived in the school’s region for a certain period yet imposes no rural service duty. Admission practices differ. Some universities administer separate exams for RQP and RQPWL, while others use a single exam for all quotas, including the general admission program (GAP). However, no reliable method has been established for selecting medical professionals who want to work in rural areas. Although financial and economic incentives have been the primary strategy for recruiting and retaining physicians in rural and remote areas, evidence of their effectiveness is mixed and relatively limited, especially in Japan [11].

Another method of recruitment is through the curriculum. In Australia, clinical training is provided at rural campuses called Rural Clinical Schools. Studies have shown that RCS graduates are more likely to practice in rural areas than their urban counterparts [12]. Introducing regulatory incentives seems useful; since 1994, the Conrad 30 Waiver Program in the United States has offered international medical school graduates (IMGs) the opportunity to waive their two-year mandatory return to their home country if they work in areas with a shortage of physicians for at least three years. The program has shown that increasing the supply of IMGs can be done sustainably without reducing the supply of physicians trained in the United States [13].

In fields other than rural medicine, several investigations have demonstrated a correlation between personality traits and the determination of employment, as well as career accomplishments within the medical field [14-16]. In personality trait research, the 'big five' or 'five-factor model' (FFM) is widely accepted. This is a theoretical model consisting of five dimensions, namely openness to experience, conscientiousness, extraversion, agreeableness, and neuroticism [17,18]. Openness to experience describes a tendency to be imaginative, creative, and curious, and to prefer variety over routine. Conscientiousness means a tendency to be hardworking, well-organized, ambitious, and persevering. Extraversion is characterized by a tendency to be affectionate, talkative, active, and fun-loving in social situations. Agreeableness relates to a tendency to be soft-hearted, trusting, generous, and good-natured in interactions with others. Neuroticism refers to a tendency to be worrying, emotional, and vulnerable, reflecting a general proneness to distress [18]. The big five personality trait model is a theory of personality systematized in the latter half of the 20th century. Widely used in psychology and various other academic and applied fields, it is considered a reliable measurement framework with excellent internal consistency and sufficient construct validity [19]. These five factors are reported to be stable factors that are observed relatively consistently across cultures and age groups and have an impact on various aspects of an individual's life [19]. Research has also shown that the FFM is useful for predicting employee job performance and career success [20]. Certain personality traits are associated with success in the careers of people with traits, such as law enforcement agencies [21] or aviation services [22].

Several studies have demonstrated that medical students’ personality tendencies can predict their future choice of specialty, especially in medicine [18-20,23,24]. However, little is known about the personality tendencies of medical students and their willingness to engage in medically underserved activities. Community health care providers are expected to do more than just treat individual patients. Their contributions are vital for addressing health disparities and improving the overall well-being of the communities they serve [25].

In Japan, community health care requires specific competencies that are developed during postgraduate training [26]. These competencies include a strong clinical focus on general or family medicine to treat a wide variety of conditions, an interest in community residents as individuals, including diverse populations such as children and the elderly, and a professional mindset characterized by flexibility, self-reliance, and intrinsic motivation. By selecting individuals who already possess these competencies, more effective educational programs could be offered.

Although previous studies have examined medical students’ personality tendencies and willingness to engage in community health care [27,28], few studies have assessed them using a validated personality model. The limited evidence base may have slowed the application of personality research to community health care. Australia shares the same challenge of increasing the number of community health care providers. Notably, a previous study from Australia has provided evidence that some individuals have a greater potential to practice in medically underserved areas, implying that certain personality traits suited to work in such settings can be identified [29]. If more robust evidence on community health care-oriented personalities is identified, it would not only advance research on personalities as the competence of the health professions, but would also provide powerful policy options for ensuring a more reliable supply of human resources for areas with physician shortages. This study aims to investigate how medical students' personality traits, gender, and enrollment status in RQPs (with or without loan obligations) are associated with (1) admission through these programs and (2) willingness to work in community health care settings.

This article was previously posted to the Research Square preprint server on April 25, 2023 (DOI: 10.21203/rs.3.rs-2827823/v1).

## Materials and methods

Study design

This is a cross-sectional study conducted at Shinshu University School of Medicine (Nagano, JPN) between October 1 and November 30, 2022. This national university, located in central Japan, has fewer physicians than Japan’s national average. There are three admission pathways available at the Shinshu University School of Medicine, consistent with the majority of medical schools in Japan. The GAP does not impose any requirements for admission to postgraduate career paths. The RQP requires applicants to be engaged in medical practice in medically underpopulated areas for at least nine years after graduation in exchange for a six-year non-refundable scholarship [30]. All participants of RQP must avail themselves of the scholarship. The efficacy of augmenting the quantity of rural physicians through the implementation of scholarship programs has been substantiated [11]. The RQPWL requires applicants to have lived in the area where the medical school is located for a certain period, but participants are not obliged to practice in medically underpopulated areas and are not required to utilize this scholarship. None of the three programs included personality assessment examinations in any manner, and no selection was made based on gender.

We employed total-population sampling, distributing an anonymous self-administered questionnaire to all first- to sixth-year students at Shinshu University School of Medicine between October and November 2022 (n=760). Inclusion criteria included enrollment as a medical student in the first through sixth year at Shinshu University School of Medicine during the 2022 academic year and physical attendance at the specific on-campus session in which the questionnaire was distributed after scheduled lectures for first- to fourth-year students, after the end of the all-in-one event for fifth-year students, and after the regular examinations for sixth-year students. The exclusion criteria were as follows: absence from the distribution session, refusal to participate, submission of a blank questionnaire, and selection of a gender category other than male or female, provided that the number of respondents in that category was too small to support reliable statistical analysis. No other exclusion criteria were applied.

Participants did not receive any incentives. Since the questionnaire was completed anonymously, the year of matriculation could not be linked to the responses. Therefore, participants are described by their current academic year (first to sixth year). All questionnaires were distributed in paper form by staff not involved in the teaching or assessment of the students.

Questionnaire

The questionnaire titled 'Questionnaire on personality traits of medical students,' in Japanese, consists of two parts. The first part consists of sociodemographic questions on gender, grade, admission method (quota), and willingness to engage in community health care (“I will do,” “I will not do,” or “not decided”). The second part consists of personality questions. Personality characteristics were measured using the shortened Japanese version of the big five personality traits as the five-factor model (FFM) [31], which consists of 29 questions. This questionnaire is a validated measure of the FFM [32], and each item is scored on a five-point Likert scale ranging from “disagree strongly” (1) to “completely agree” (5). The items correspond to neuroticism, extraversion, openness to experience, conscientiousness, and agreeableness and consist of five, five, six, six, and seven questions, respectively. Therefore, the score ranges are 0-25, 0-25, 0-30, 0-30, and 0-35 points, respectively. An English translation of the questionnaire items and the corresponding table for each scale are given in Appendix A.

Two faculties who work for community practice, a medical education researcher (the last author), and our research team collaborated to develop this questionnaire based on prior studies regarding the validity and reliability of FFM [31]. There are many versions of the FFM questionnaire, including a 10-item shortened version and a 300-item questionnaire. Long questionnaires are highly reliable, but they place a heavy burden on respondents and may result in low response rates. On the other hand, short questionnaires may not be reliable. Considering these advantages and disadvantages, we ultimately adopted the 29-item questionnaire. In the original publication, the scale demonstrated acceptable to good internal consistency: Cronbach's alpha was 0.82 for neuroticism, 0.86 for extraversion, 0.76 for openness to experience, 0.78 for conscientiousness, and 0.78 for agreeableness.

Statistical analysis

Descriptive statistics for the variables in the first part were calculated for all the respondents. Next, the scale scores for each factor of the FFM were calculated from the second part of the questionnaire, which includes all questions of the shortened Japanese versions of the big five personality traits. Cronbach’s alpha was calculated to assess the internal consistency of responses, and Spearman’s correlation coefficient was calculated for each factor. Respondents were divided into three groups based on the type of entrance examination: RQP, RQPWL, and GAP. Comparisons between groups were made using Welch’s test, the Kruskal-Wallis test, Fisher’s exact test, or Pearson’s chi-square test, depending on the basic index and distribution of the dependent variable.

Next, to evaluate the relationship between the admission method and personality, we conducted four multiple logistic regressions. The dependent variable was whether the admission method was RQP/RQPWL, and the control group was GAP. The independent variables were either (1) each factor of the FFM or (2) each question item in the second part.

Finally, a multiple logistic regression was conducted to evaluate the association between willingness to engage in community health care and personality. The dependent variable was the willingness to engage in community health care. The independent variables were sex, grade, admission method, and either (1) each factor of the FFM or (2) each item of the second part of the questionnaire. In the analysis of the 'willingness to engage in community health care' as the dependent variable, “not decided” responses were treated as “no motivation,” hence the responses were binary.

In the series of logistic regressions, the analysis using the FFM factors is the main analysis, and the analysis using each item of the second part of the questionnaire is an exploratory analysis for hypothesis generation. Upon examination of each item of the second part of the questionnaire, a univariate logistic regression was initially employed to identify items with a p-value less than 0.25. Subsequently, variables were chosen based on the Akaike information criterion (AIC), utilizing a bootstrap procedure on a data set of 1000 resamples. The variables retained in at least 50% of the iterations were included in the final regression model [33]. The results are summarized as odds ratios (ORs) and 95% confidence intervals (CIs), and model fitness was checked using the Hosmer-Lemeshow test.

To evaluate the effect of including quota-by-gender interaction terms in the model, we performed a likelihood ratio test. We entered quotas (GAP, RQP, and RQPWL), gender, and a quota-by-gender interaction as covariates, along with academic year and FFM factors. We used a likelihood-ratio test to screen for effect modification by comparing models with and without the interaction. Then we estimated gender-specific adjusted odds ratios for each quota group with the emmeans package [34]. Pairwise quota contrasts were multiplicity-adjusted using the Tukey-Kramer method.

As a robustness check, we also fitted a multinomial logistic regression model with three outcome categories (“I will do,” “I will not do,” or “not decided”) to test whether collapsing “I will not do” and “not decided” responses altered the results. We entered the same covariates into the model, using “I will not do” as the reference category. Model fit was evaluated with a likelihood-ratio test and summarized with Nagelkerke’s R².

Since the questionnaire was distributed to the entire student body of 760 students, the final sample size was determined by the response rate rather than a priori power considerations. We prespecified that we would assess model stability after data collection using the events-per-variable (EPV) rule of Peduzzi et al. [35], which recommends at least 10 outcome events for each regression coefficient in a logistic model; models below this threshold were interpreted as exploratory.

All statistical tests were performed at a significance level of 0.05. Statistical analyses were performed in R version 4.4.2 (R Foundation for Statistical Computing, Vienna, AUT) with the Comprehensive R Archive Network (CRAN) MASS package [36] (version 7.3-61; stepAIC function), the bootStepAIC package [37] (version 1.3-0; boot.stepAIC function), and the emmeans package [34] (version 1.11.1; emmeans function).

Ethical considerations

Students received a written informed consent form, and all the participants were briefed that the survey was not mandatory and would not be used for other purposes such as their grading or their future career mentorings. Those who understood the purpose of the research and agreed to participate received the questionnaires. Those who understood the purpose of the research and agreed to participate returned the completed questionnaire form as consent to the participation. The participants are adults over the age of 18. The study was approved by the Ethics Review Board of Shinshu University School of Medicine (approval no. 5626).

## Results

Of the 750 students, 601 (80.1%) responded from the first to the sixth year (37.2% female). In the analysis, six respondents (0.99%) had at least one unanswered item in the second part of the questionnaire. We examined the pattern of missingness and found no association with key baseline variables (gender, quota, grade, or willingness; all Fisher's exact tests p > 0.20), suggesting the data were missing completely at random (MCAR). Given the very small proportion (< 1 %) and the MCAR pattern, we excluded these cases listwise from the multivariable analyses. Four respondents who identified their gender as “other” were excluded from the analysis. This was made for two reasons. One, primarily because the study aimed to investigate the relationship between binary gender categories and admission methods. Two, the small number of participants in this group led to insufficient statistical power, potentially affecting the validity and reliability of our analyses. Consequently, the final sample used for analysis consisted of 591 (78.8%) participants (37.2% female). The breakdown of the respondents by admission methods is presented in Table 1.

**Table 1:** The number of respondents from the three programs RQP: Regional quota program, RQPWL: Regional quota program without loan, GAP: General admission program

Quota	Male (n=371)	Female (n=220)	Total
RQP	41	36	77 (13.0%)
RQPWL	16	23	39 (6.6%)
GAP	314	161	475 (80.4%)

Five factors were calculated from the second section of the questionnaire: extraversion (E), openness to experience (O), agreeableness (A), conscientiousness (C), and neuroticism (N). The total Cronbach’s alpha value for the second part of the questionnaire was 0.84, indicating a good level of internal consistency. Cronbach’s alpha values for the five factors were as follows: 0.87 (E), 0.81 (O), 0.78 (A), 0.83 (C), and 0.82 (N), respectively. The correlations among FFM values are listed in Table 2.

**Table 2:** Correlation among the FFM values Cronbach’s alpha: Extraversion 0.87, openness to experience 0.81, agreeableness 0.78, conscientiousness 0.83, neuroticism 0.82. Upper triangle entries are Spearman’s correlation coefficients. Lower triangle entries give the test statistics and p-values. Bold font indicates statistical significance. FFM: Five-factor model

Extraversion	Openness to experience	Agreeableness	Conscientiousness	Neuroticism
-	0.358	0.041	0.010	-0.363
p<0.001, t=9.312	-	0.151	0.161	-0.291
p=0.317, t=1.001	p<0.001, t=3.700	-	0.204	-0.139
p=0.807, t=0.245	p<0.001, t=3.956	p<0.001, t=5.058	-	-0.002
p<0.001, t=-9.461	p<0.001, t=-7.376	p<0.001, t=-3.397	p=0.953, t=-0.059	-

Regarding sociodemographic questions, a significant difference was found in admission methods between the genders; the ratio of male to female participants differed between RQP, RQPWL, and GAP (Fisher’s exact test, p = 0.002). There were no differences in four of the five factors of the FFM questionnaire when comparing the group based on their gender: extroversion (Welch’s t-test, t = -0.48, p = 0.63), agreeableness (Welch’s t-test, t = 0.59, p = 0.56), conscientiousness (Welch’s t-test, t = 0.16, p = 0.87), and neuroticism (Welch’s t-test, t = -0.74, p = 0.46). A significant difference was found in openness to experience (Welch’s t-test, t = 3.4, p < 0.001). The mean level of openness to experience was 18.29 (SD = 4.54) for men and 16.93 (SD = 4.72) for females. The results are shown in Table 3. Next, the responses to 'willingness to engage in community health care' were divided into three groups: “I will,” “I will not,” and “not decided” (Table 4). Female students were more willing to engage in community health care than males (Fisher’s exact test, p = 0.016). There was a significant difference in the willingness to engage in community health care between the quotas (Fisher’s exact test, p < 0.001).

**Table 3:** Charactereistics broken down by gender The personality items in the table represent means, and those in parentheses represent standard deviations. For the categorical value, i.e., quota, Fisher’s exact test was used; no test statistic is reported, as the test is based on exact probabilities rather than a test statistic (shown as N/A). Bold font indicates statistical significance. RQP: Regional quota program, RQPWL: Regional quota program without loan, GAP: General admission program

Variable		Male	Female	Statistic	p-value
Quota	RQP	41	36	N/A	0.002
	RQPWL	16	23		
	GAP	314	161		
Personality	Extraversion	14.64 (SD 4.01)	14.81 (SD 4.18)	t=-0.48	0.63
	Openness to experience	18.29 (SD 4.54)	16.93(SD 4.72)	t=3.4	<0.001
	Agreeableness	17.44 (SD 3.96)	17.24 (SD 4.19)	t=0.59	0.56
	Conscientiousness	14.32 (SD 5.31)	14.25 (SD 5.11)	t=0.16	0.87
	Neuroticism	16.32 (SD 4.25)	16.60 (SD 4.55)	t=-0.74	0.46

**Table 4:** Characteristics broken down by willingness to do community health care Parentheses in gender indicate the percentage in each gender. Parentheses in quota indicate the percentage for the combination of quota and response. The personality items in the table represent means, and those in parentheses represent standard deviations. For categorical variables such as gender and quota, Fisher’s exact test was used; no test statistic is reported, as the test is based on exact probabilities rather than a test statistic (shown as N/A). Bold font indicates statistical significance. RQP: Regional quota program, RQPWL: Regional quota program without loan, GAP: General admission program

Variable		I will	I will not	Not decided	Statistic	p-value
Gender	Male	112 (30.1%)	114 (30.70%)	145 (39.10%)	N/A	0.016
	Female	92 (41.80%)	59 (26.80%)	69 (31.40%)		
Quota	RQP	67 (11.30%)	3 (0.50%)	7 (1.20%)	N/A	<0.001
	RQPWL	24 (4.10%)	2 (0.30%)	13(2.20%)		
	GAP	113 (19.10%)	168 (28.40%)	194 (32.80%)		
Personality	Extraversion	14.10 (SD 4.16)	14.64 (SD 3.97)	15.51 (SD 3.98)	H(2)=12.519	0.002
	Openness to experience	17.96 (SD 4.90)	17.97 (SD 4.88)	17.46 (SD 4.20)	H(2)=0.885	0.642
	Agreeableness	17.788 (SD 4.28)	16.71 (SD 3.91)	17.41 (SD 3.87)	H(2)=9.240	0.01
	Conscientiousness	14.77 (SD 5.55)	14.00 (SD 5.19)	14.07 (SD 4,94)	H(2)=2.787	0.248
	Neuroticism	16.74 (SD 4.45)	16.06 (SD 4.28)	16.41 (SD 4.35)	H(2)=2.486	0.289

There were no differences in three of the five factors of the FFM questionnaire when comparing the groups based on their willingness to engage in community health care: openness to experience (Kruskal-Wallis Test, H(2) = 0.885, p = 0.64), conscientiousness (Kruskal-Wallis Test, H(2) = 2.787, p=0.25), and neuroticism (Kruskal-Wallis Test, H(2) = 2.486, p=0.29). Significant differences were found for extraversion (Kruskal-Wallis Test, H(2) = 12.519, p = 0.002) and agreeableness (Kruskal-Wallis Test, H(2) = 9.240, p = 0.010).

Logistic regression analysis on admission method(s)

Regional Quota Without Loan

Tables 5-6 illustrate the analysis of the RQPWL (n=514) with the GAP as a control. The p-values of the Hosmer-Lemeshow test were 0.626 (Table 5) and 0.396 (Table 6), respectively, indicating a good fit. Regarding personality (Table 5), grade and gender were significant. In relation to the question items (Table 6), eight variables (Q2-4, Q12, Q14, Q15, Q18, Q21), grade, and gender met the initial screening criterion (p < 0.25). Among these, Q2, Q21, grade, and gender were significant (Table 6). The complete list of screened variables is provided in Appendix B.

**Table 5:** Logistic regression analysis between RQPWL and FFM factors (n=514) Bold font indicates statistical significance. RQPWL: Regional quota program without loan, FFM: Five-factor model

Variable	Statistic	p-value	Adjusted OR (95% CI)
Grade (1–6)	z=-2.543	0.011	0.77 (0.63–0.94)
Gender: Female (ref: male)	z=3.058	0.002	2.94 (1.48–5.96)
Extraversion	z=-1.944	0.052	0.91 (0.83–1.00)
Openness to experience	z=1.911	0.056	1.08 (0.998–1.17)
Agreeableness	z=0.009	0.993	1.00 (0.92–1.09)
Conscientiousness	z=0.882	0.378	1.03 (0.96–1.10)
Neuroticism	z=1.400	0.161	1.6 (0.98–1.16)

**Table 6:** Logistic regression analysis between RQPWL and question items of the second part (FFM questions; n=514) Bold font indicates statistical significance. RQPWL: Regional quota program without loan, FFM: Five-factor model

Variable	Statistic	p-value	Adjusted OR (95% CI)
Grade	z=-2.319	0.020	0.79 (0.64-0.96)
Gender: Female (ref: male)	z=2.943	0.003	2.82 (1.42–5.72)
Q2) Sociable	z=-2.706	0.007	0.60 (0.42–0.87)
Q21) Sharp-witted	z=2.688	0.007	1.58 (1.13–2.22)

Regional Quota Program

Tables 7-8 illustrate the analysis of RQP (n=552) in contrast to GAP. The p-values of the Hosmer-Lemeshow test were 0.645 (Table 7) and 0.891 (Table 8), respectively, indicating a good fit. Extraversion, conscientiousness, and gender were significantly associated with personality (Table 7). Regarding the questionnaire items, 16 items (Q1-Q7, Q10-12, Q14, Q19-21, Q25-26) and gender met the initial screening criterion (p < 0.25). Among these, Q6 and Q20 were significant (Table 8). The complete list of screened variables is provided in Appendix C.

**Table 7:** Logistic regression analysis between RQP and FFM factors (n=552) Bold font indicates statistical significance. RQP: Regional quota program, FFM: Five-factor model

Variable	Statistic	p-value	Adjusted OR (95% CI)
Grade (1–6)	z=-0.253	0.800	0.98 (0.85–1.13)
Gender (men/women)	z=2.201	0.028	1.76 (1.06–2.92)
Extraversion	z=-2.527	0.012	0.91 (0.85–0.98)
Openness to experience	z=-0.422	0.673	0.99 (0.93–1.05)
Agreeableness	z=0.781	0.435	1.03 (0.96–1.10)
Conscientiousness	z=1.963	0.0496	1.05 (1.00–1.10)
Neuroticism	z=0-0.227	0.820	1.99 (0.93–1.06)

**Table 8:** Logistic regression analysis between RQP and question items of the second part (FFM questions; n=552) Bold font indicates statistical significance. RQP: Regional quota program, FFM: Five-factor model

Variable	Statistic	p-value	Adjusted OR (95% CI)
Gender: Female (ref: male)	z=1.546	0.122	1.48 (0.90–2.42)
Q6) Irresponsible	z=2.713	0.007	1.38 (1.09–1.75)
Q20) Original	z=-2.489	0.013	0.74 (0.58–0.93)

Logistic Regression Analysis on Willingness to Engage in Community Healthcare

The results of the multivariate logistic regression analysis of the FFM factors are presented in Table 9. The p-value of the Hosmer-Lemeshow test was 0.500, indicating a good fit. There was a strong association between willingness to engage in community healthcare for students enrolled in the RQP (OR 20.69, 95% CI 10.64-44.38, p < 0.001) and those enrolled in the RQPWL (OR 4.32, 95% CI 2.16-8.90, p < 0.001). Regarding personality, there was a significant association between willingness to engage in community healthcare for openness to experience (OR 1.06, 95% CI 1.01-1.11, p = 0.027). There was also a significant but negative association with extraversion (OR 0.94, 95% CI 0.89-1.00, p = 0.046). A weakly significant association at the p < 0.1 level was observed for agreeableness (OR 1.05, 95% CI 1.00-1.11, p = 0.054). There was a significant association with gender (OR 1.57, 95% CI 1.04-2.36, p = 0.031). On the other hand, grade was not associated with willingness to engage in community healthcare.

**Table 9:** Logistic regression analysis between FFM factors and willingness to engage in community health care Bold font indicates statistical significance. RQP: Regional quota program, RQPWL: Regional quota program without loan, FFM: Five-factor model

Variable	Statistic	p	Adjusted OR (95% CI)
Grade (1–6)	z=0.200	0.842	1.01 (0.90–1.13)
Gender: Female (ref: Male)	z=2.154	0.031	1.57 (1.04–2.36)
RQPWL	z=4.076	<0.001	4.32 (2.16–8.90)
RQP	z=8.400	<0.001	20.69 (10.65–44.38)
Extraversion (E)	z=-1.999	0.046	0.94 (0.89–1.00)
Openness to experience (O)	z=2.219	0.027	1.06 (1.01–1.11)
Agreeableness (A)	z=1.928	0.054	1.05 (1.00–1.11)
Conscientiousness (C)	z=-0.064	0.949	1.00 (0.96–1.04)
Neuroticism (N)	z=0.714	0.475	1.02 (0.97–1.07)

Regarding the questionnaire items, 17 items (Q1-7, Q9, Q11, Q14, Q16, Q22-27) and gender met the initial screening criterion (p < 0.25). The complete list of screened variables is provided in Appendix D. The p-value of the Hosmer-Lemeshow test was 0.814, indicating a good fit. There was also a strong association between willingness to engage in community health care for students enrolled in the RQP (OR 20.91, 95% CI 10.80-44.72, p < 0.001) and those enrolled in the RQPWL (OR 5.13, 95% CI 2.59-10.46, p < 0.001). For each item, Q4, Q23, and Q24 were significant for willingness to engage in community health care (Table 10).

**Table 10:** Logistic regression analysis between question items of the second part and willingness to engage in community health care Bold font indicates statistical significance. RQP: Regional quota program, RQPWL: Regional quota program without loan

Variable	Statistic	p-value	Adjusted OR (95% CI)
RQPWL	z=4.623	<0.001	5.13 (2.59–10.46)
RQP	z=8.468	<0.001	20.91 (10.80-44.72)
Q4) Outgoing	z=-2.308	0.021	0.79 (0.64–0.96)
Q23) Curious	z=2.605	0.009	1.30 (1.07–1.59)
Q24) Impatient	z=2.032	0.042	1.20 (1.01–1.43)

Interaction analysis on quota and gender

The multivariable logistic regression model, which included quota (GAP, RQP, and RQPWL), gender, the prespecified quota-by-gender interaction, academic year, and FFM factors, showed a good fit (Hosmer-Lemeshow, p = 0.597). The likelihood-ratio test confirmed that the quota-by-gender interaction improved model fit (χ² = 14.9, 2 degrees of freedom, p < 0.001), showing that quota effects differ by sex. Among women the RQP and RQPWL both showed a pronounced advantage over the GAP: relative to GAP, the odds of expressing willingness were 14.6 (95% CI 4.63-46.0, p < 0.001) for RQP and 17.7 (95% CI 3.83-81.5, p < 0.001) for RQPWL, and the two quotas did not differ from each other (OR 0.83, 95% CI 0.14-5.04, p = 0.967).

For men, a different pattern emerged. RQP produced a very large contrast with GAP (OR 30.74, 95% CI 8.52-110.9, p < 0.001), but RQPWL produced virtually no contrast at all (OR 1.00, 95% CI 0.24-4.12 versus GAP, p = 1.000). Consequently, when we compare the two regional admission quotas directly, their effect differs sharply by gender: in women, the difference between RQP and RQPWL is negligible, whereas in men, the RQP yields odds about 31 times higher than the RQPWL. These results show a clear gender contrast: females respond strongly to RQPWL, matching the effect of RQP, whereas males respond only under RQP and show no change at all under RQPWL (Table 11).

**Table 11:** Odds of expressing willingness compared to other quota, by gender Bold font indicates statistical significance. RQP: Regional quota program, RQPWL: Regional quota program without loan, GAP: General admission program

Contrast		Statistic	p-value	Adjusted OR (95% CI)
Male	RQPWL/GAP	0.001	1.000	1.00 (0.24-4.12)
	RQP/GAP	6.256	<0.001	30.74 (8.52-110.9)
	RQP/RQPWL	4.327	<0.001	30.71 (4.81-196.3)
Female	RQPWL/GAP	4.402	<0.001	17.66 (3.83-81.5)
	RQP/GAP	5.471	<0.001	14.58 (4.63-46.0)
	RQP/RQPWL	-0.249	0.967	0.83 (0.14-5.04)

Sample-size adequacy analysis

Table 12 summarizes the events-per-variable (EPV) for the six logistic regression analyses. The EPV ratios ranged from 5.6 to 40.8 across the models. Four of the six analyses met the recommended threshold of ten events per predictor variable [35]. The RQPWL models showed EPV ratios of 5.6 for the FFM and 9.8 for the question items. The FFM model fell below the recommended threshold. The RQP models demonstrated EPV ratios of 11.0 (FFM) and 25.7 (question items); both exceeded the threshold. The willingness to engage in community health care models exhibited the highest EPV ratios, at 22.7 (FFM) and 40.8 (question items), indicating adequate model stability.

**Table 12:** The EPV summary for logistic models RQWL: Regional quota without loan, RQP: Regional quota program, FFM: Five-factor model

Logistic regression analyses	Factors	Number of events	Number of variables	EPV
RQWL	FFM	39	7	5.6
RQWL	Question	39	4	9.8
RQP	FFM	77	7	11.0
RQP	Question	77	3	25.7
Willingness to engage in community health care	FFM	204	9	22.7
Willingness to engage in community health care	Question	204	5	40.8

Robustness check for the willingness outcome using a multinomial model with three response categories

A likelihood-ratio test confirmed that the multinomial specification fit the data substantially better than an intercept-only model (χ² = 161.92, df = 18, p < 0.0001), and the model explained about 27% of the variance (Nagelkerke R² = .27). The results of the multinomial logistic regression analysis are presented in Appendix E. Each variable is listed twice. The first listing shows the contrast between "Undecided" and "I will not," and the second listing shows the contrast between "I will" and "I will not." The multinomial robustness check largely reproduced the directional pattern observed in the primary binary model (Table 9). In both models, the two quota indicators were strongly and positively associated with willingness (p < 0.001 for each). Higher scores on extraversion were again associated with lower odds of willingness (p < 0.05 in both models). The openness to experience variable retained a positive coefficient and reached conventional significance in the binary model (p = 0.027) while remaining at the trend level in the multinomial model (p = 0.092). Agreeableness displayed the opposite pattern: a positive trend in the binary model (p = 0.054) and full significance in the multinomial model (p = 0.011). Gender was not significant, though its positive sign matched the binary model. Thus, aside from the loss of statistical significance for gender, the direction and strength of the effects of personality and quotas were consistent. This indicates that the key findings are stable even when the outcome is estimated with three categories.

## Discussion

In this study, the personality profiles of students inclined toward community-oriented medicine and those not inclined toward community-oriented medicine were compared using a logistic regression when examining orientation toward community-oriented medicine. In addition, each RQP and RQPWL was investigated using a logistic regression and compared to the GAP. This study is groundbreaking because it uses the commonly used FFM to clarify personality tendencies and willingness to engage in community-oriented medicine among medical students.

The results of our study indicate that women are more likely to enter medical school through the RQPWL or RQP quotas. These results suggest that, in Japan, women living in rural prefectures are more likely to behave in ways that remain within the prefecture. In fact, surveys of women in rural areas have shown that women tend to stay in rural areas due to work-life balance and family ties [38,39]. Some surveys show that women place importance on family ties when choosing where to live [40]. In Japan, pursuing an academic career in the medical field is frequently regarded as difficult for women. Kono et al. demonstrated that female physicians exhibit lower academic accomplishments in Japan [41]. The academic trajectories of physicians in Japan are frequently influenced by their associations with urban-based universities, with a distinct delineation between community-oriented medicine and scholarly pursuits. As a result, some female students may tend to avoid specialized scholarship and choose the practice of community-oriented medicine. The ratio of male to female medical students is high in Japan currently [42]. Addressing gender imbalance by recruiting more female students and providing them with adequate career support may change the current career choices.

Our results demonstrated that the personality characteristics between the RQP and community medicine-oriented groups clearly differed in the correlations between the elements of FFM and several questions, and this indicated that the two cohorts were different. It seems that some of the students who entered medical school through RQP have no intention of practicing community-oriented medicine but rather take advantage of the financial incentive of receiving a scholarship loan that does not have to be repaid. Kataoka et al. surveyed students who entered Japanese university medical schools through regional quotas and found that approximately 40% of them may repay their scholarships [43]. Repaying scholarships suggests a refusal to work in regional healthcare. It is possible that the attempt to increase the number of medical personnel engaged in community-oriented medicine by providing financial incentives may be a cause of the mismatch and that the most appropriate personnel may not be selected. About the requirement to work in medically underserved areas, mandatory interventions such as a commitment to practice in a rural area have a lower regional retention rate than less mandatory interventions such as the selection of university students with rural backgrounds and providing rural training during university and postgraduate education [44], and as a result, the number of health professionals working in community health may not increase.

According to these data, we can infer that the current selection of medical students by the RQP may not adequately pick up on the students who want to engage in community-oriented medicine. However, it was set up to increase the number of doctors involved in community-oriented medicine. Therefore, it is necessary to further optimize the entrance examination format to increase students’ interest in engaging in community-oriented medicine.

The results illustrated that the community-medicine-oriented group demonstrated a positive correlation between openness to experience and agreeableness and a negative correlation with extraversion. Regarding the high score of agreeableness, we interpreted that those who have the ability to get along with others well are inclined toward community-oriented medicine because it is essential in community-oriented medicine to conduct medical care tailored to the community while keeping oneself in harmony with others.

Furthermore, in the group inclined toward community-oriented medicine, there was a positive correlation with openness, which is sometimes interpreted as reflecting intellect. A meta-analysis has shown that openness exhibits the strongest correlation with general intelligence [45,46]. Population aging is progressing much faster in rural areas than in metropolitan areas of Japan. As a result, these regions are expected to face various problems earlier and more severely than the rest of the country. Therefore, those with openness may be considered a group willing to work on solving such issues. Openness is a factor that predicts training proficiency regardless of occupation [20,47], and it is thought to be appropriate for community health care, which involves extensive and often difficult tasks [48]. From the viewpoint of novelty seeking, the relevance of openness becomes even clearer: physicians working in community settings must often provide care with limited resources, develop new solutions, and treat patients of all ages with a wide range of diseases, forcing them to learn unfamiliar areas of medicine. Openness to experience can therefore be put to practical use in meeting these novel challenges.

Finally, there was a negative correlation with extraversion in the group inclined toward community-oriented medicine. It has been reported that the more extroverted a person is, the more likely he or she is to live in an urban area [49,50]. In fact, university students in urban areas show higher levels of extraversion than those in rural areas [51]. It seems that regional orientation is different from urban orientation. There was a positive correlation between openness and extraversion, similar to that found in the previous large-scale survey in Japan [52]. Highly extroverted people tend to seek new stimuli, which is related to openness's novel-seeking trait. Royston et al. found that students with high extraversion were more likely to want to practice medicine in rural areas [28]. By contrast, in our data, willingness to engage in community-oriented medicine was positively associated with openness to experience but was greater among less extroverted (i.e., more introverted) students.

We believe it is crucial to admit students with these personality traits to increase the number of medical professionals engaged in community-oriented medicine. For example, applicants with high extraversion scores may be mismatched with regard to the provision of community health care, and adjustments may be necessary to prevent this from occurring. For selective admission of students with these personality traits, it may be effective, for instance, to conduct multiple mini-interviews during the admission interview and to provide a task that looks at these personality traits. One study demonstrated the usefulness of multiple mini-interviews in selecting physician assistant (PA) programs in the United States [53]. In Japan, where university entrance exams traditionally emphasize equality and fairness, introducing character assessments requires caution. Nevertheless, the Ministry of Education, Culture, Sports, Science and Technology (MEXT) advises universities to “comprehensively evaluate the abilities and aptitudes necessary for students to study and graduate” [54] when admitting students. This guidance permits holistic assessments as long as the criteria are clearly defined, standardized, and consistent with each institution’s mission. Structured, validated, and transparent tools are strongly recommended for assessing noncognitive attributes such as commitment to community health care.

Although FFM is known to be highly stable [55,56], it can change due to life events [57], development [58]. It is said to become more stable with age, so there is a possibility that it will change during adolescence [59,60]. In particular, there have been cases in which interventions changed the personality tendencies of primary care physicians [61], and it has been suggested that early work experience is related to personality development in young adulthood [57]. Alessandri et al. state that personality changes during the process of organizational socialization of young people [62]. This indicates that personality development takes place during the process of organizational socialization in medical training. In other words, it suggests the possibility of interventions to increase the orientation towards community-oriented medicine during the process of medical training. For example, it is possible to propose the introduction of collaborative learning in medical education to develop a tendency toward agreeableness. This educational method will enable medical students to not only learn medicine but also develop their interactive communication and cooperation, and as a result, may increase the number of students who want to practice medicine in the community [63].

This study has several strengths. First, we achieved an 80% response rate from a diverse range of grades, from first- to sixth-year students. This reduces non-response bias. Second, we used a 29-item Japanese short form of the Big Five Inventory that has been validated. The internal consistency of this inventory in our sample was excellent (Cronbach’s alpha = 0.84). Third, including both RQP/RQPWL and GAP admission groups enabled direct comparisons under real-world admission policies.

Limitations

This study has several limitations. First, the data were collected cross-sectionally at a single time point. Therefore, it is unclear whether students who indicated their intention to engage in community healthcare chose to pursue a career in community healthcare. Consequently, causal relationships cannot be inferred, and the findings should be interpreted as associations between variables. Second, all data were obtained through self-reported questionnaires, which may have introduced response bias. Third, this study was conducted at a single facility in a single region; therefore, the generalizability of the results should be carefully considered. It has been reported that in Japan, there are regional differences in personality traits [64]. Additionally, cultural differences between countries may limit the international applicability of our results. Therefore, studies on institutions in other regions are desirable. Fourth, we must acknowledge that the effects on willingness to engage in community health care for our study subjects’ five main personality traits were small, which may limit the practical application of our results to a certain extent. Fifth, our research focused on students' intentions to serve as healthcare providers in medically underserved areas upon their admission to medical school. We did not evaluate how these intentions might change throughout medical school education. Future research could explore this aspect further. Lastly, the relationship between career intentions and personality traits has not been clearly established in the literature, possibly due to measurement difficulties. Future research may address this gap.

## Conclusions

Implementing measures to rectify gender diversity and carefully choosing medical students with a demonstrated interest in rural practice can provide a highly efficacious solution. In addition, incorporating coordination education into the medical school curriculum has the potential to significantly bolster the number of medical graduates dedicated to providing care in underserved rural areas. This research may contribute to identifying potential future medical providers who intend to practice in community-oriented and rural settings.

## Appendices

Appendix A

The English translation of the second part of the questionnaire is presented in Table 13.

**Table 13:** English translation of the second part of the questionnaire Respondents were instructed to answer the following question before taking the survey: "How well do the following statements describe your personality?" The respondents were also asked to rate each word listed in the table on a scale of 1 to 5 to indicate how true it is and to select the appropriate option.

I see myself as ….	Disagree strongly	Disagree a little	Neither agree not disagree	Agree a little	Completely agree
Q1) Silent	1	2	3	4	5
Q2) Sociable	1	2	3	4	5
Q3) Talkative	1	2	3	4	5
Q4) Outgoing	1	2	3	4	5
Q5) Cheerful	1	2	3	4	5
Q6) Irresponsible	1	2	3	4	5
Q7) Careless	1	2	3	4	5
Q8) Spontaneous	1	2	3	4	5
Q9) Lazy	1	2	3	4	5
Q10) Deliberate	1	2	3	4	5
Q11) Hasty	1	2	3	4	5
Q12) Methodical	1	2	3	4	5
Q13) Unstable	1	2	3	4	5
Q14) Worrying	1	2	3	4	5
Q15) Timid	1	2	3	4	5
Q16) Tense	1	2	3	4	5
Q17) Gloomy	1	2	3	4	5
Q18) Versatile	1	2	3	4	5
Q19) Progressive	1	2	3	4	5
Q20) Original	1	2	3	4	5
Q21) Sharp-witted	1	2	3	4	5
Q22) Wide interests	1	2	3	4	5
Q23) Curious	1	2	3	4	5
Q24) Impatient	1	2	3	4	5
Q25) Touchy	1	2	3	4	5
Q26) Mild	1	2	3	4	5
Q27) Generous	1	2	3	4	5
Q28) Egoistical	1	2	3	4	5
Q29) Kind	1	2	3	4	5

Table 14 features the question items of the second part of the questionnaire.

**Table 14:** Breakdown of the question items of the second part of the questionnaire

Factors	Question Numbers	Inverted items
Extraversion	Q1, Q2, Q3, Q4, Q5	Q1
Openness to experience	Q18, Q19, Q20, Q21, Q22, Q23	N/A
Agreeableness	Q24, Q25, Q26, Q27, Q28, Q29	Q24, Q25, Q28
Conscientiousness	Q6, Q7, Q8, Q9, Q10, Q11, Q12	Q6, Q7, Q8, Q9, Q11
Neuroticism	Q13, Q14, Q15, Q16, Q17	N/A

Appendix B

Table 15 lists the predictors that met the univariate screening criterion for admission to the RQPWL.

**Table 15:** Predictors that met the univariate screening criterion (p < 0.25) for admission through the RQPWL Bold font indicates statistical significance. RQPWL: Regional quota program without loan

Variable	Statistic	p-value	Adjusted OR (95% CI)
Grade	z=-2.557	0.011	0.78 (0.65-0.94)
Gender: Female (ref: male)	z=2.821	0.004	2.59 (1.33–5.02)
Q2) Sociable	z=-2.203	0.028	0.68 (0.48-0.96)
Q3) Talkative	z=-1.840	0.007	0.74 (0.54-1.02)
Q4) Outgoing	z=-1.450	0.147	0.78 (0.56-1.09)
Q12) Methodical	z=1.490	0.136	1.26 (0.93-1.71)
Q14) Worrying	z=1.910	0.056	1.36 (0.99-1.88)
Q15) Timid	z=1.704	0.088	1.30 (0.96-1.75)
Q18) Versatile	z=1.385	0.166	1.23 (0.92-1.64)
Q21) Sharp-witted	z=1.366	0.172	1.24 (0.91-1.70)

Appendix C 

Table 16 features predictors that met the univariate screening criterion (p < 0.25) for admission through the RQP.

**Table 16:** Predictors meeting the univariate screening criterion (p < 0.25) for admission through the RQP Bold font indicates statistical significance. RQP: Regional quota program

Variable	Statistic	p-value	Adjusted OR (95% CI)
Gender: Female (ref: male)	z=1.844	0.065	1.57 (0.97-2.55)
Q1) Silent	z=-2.348	0.019	0.76 (0.61-0.96)
Q2) Sociable	z=-2.125	0.034	0.76 (0.59-0.98)
Q3) Talkative	z=-1.449	0.147	0.84 (0.66-1.06)
Q4) Outgoing	z=-2.507	0.012	0.73 (0.57-0.93)
Q5) Cheerful	z=-1.979	0.048	0.79 (0.62-1.00)
Q6) Irresponsible	z=2.526	0.012	1.34 (1.07-1.69)
Q7) Careless	z=2.587	0.010	1.32 (1.07-1.64)
Q10) Deliberate	z=1.336	0.180	1.17 (0.93-1.46)
Q11) Hasty	z=1.857	0.063	1.24 (0.99-1.56)
Q12) Methodical	z=1.200	0.230	1.15 (0.92-1.43)
Q14) Worrying	z=1.491	0.136	1.18 (0.64-1.03)
Q19) Progressive	z=-1.731	0.083	0.81 (0.64-1.03)
Q20) Original	z=-2.590	0.096	0.73 (0.58-0.93)
Q21) Sharp-witted	z=-1.170	0.242	0.87 (0.69-1.10)
Q25) Touchy	z=1.436	0.151	1.19 (0.94-1.50)
Q26) Mild	z=1.176	0.239	1.18 (0.90-1.54)

Appendix D

Table 17 features the predictors that met the univariate screening criterion (p < 0.25) for willingness to engage in community healthcare.

**Table 17:** Predictors that met the univariate screening criterion (p < 0.25) for willingness to engage in community healthcare Bold font indicates statistical significance. RQWL: Regional quota without loan, RQP: Regional quota program

Variable	Statistic	p-value	Adjusted OR (95% CI)
Gender: Female (ref: male)	z=2.864	0.004	1.66 (1.17-2.35)
RQP	z=8.247	<0.001	18.44 (9.22-36.85)
RQPWL	z=3.503	<0.001	3.31 (1.69-6.46)
Q1) Silent	z=-1.688	0.091	0.87 (0.74-1.02)
Q2) Sociable	z=-2.819	0.005	0.77 (0.65-0.92)
Q3) Talkative	z=-1.362	0.173	0.89 (0.75-1.05)
Q4) Outgoing	z=-2.935	0.062	0.77 (0.65-0.92)
Q5) Cheerful	z=-1.865	0.003	0.85 (0.72-1.01)
Q6) Irresponsible	z=1.692	0.091	1.15 (0.98-1.36)
Q7) Careless	z=2.055	0.04	1.17 (1.01-1.36)
Q9) Lazy	z=1.489	0.136	1.13 (0.96-1.32)
Q11) Hasty	z=2.550	0.011	1.23 (1.05-1.45)
Q14) Worrying	z=1.675	0.094	1.14 (0.98-1.33)
Q16) Tense	z=1.651	0.099	1.14 (0.98-1.34)
Q22) Wide interests	z=1.622	0.105	1.12 (0.98-1.29)
Q23) Curious	z=1.733	0.083	1.16 (0.98-1.37)
Q24) Impatient	z=2.123	0.034	1.18 (1.01-1.38)
Q25) Touchy	z=1.897	0.058	1.17 (0.99-1.38)
Q26) Mild	z=2.118	0.034	1.23 (1.02-1.49)
Q27) Generous	z=1.363	0.173	1.14 (0.94-1.38)

Appendix E

The results of the multinomial logistic regression analysis are presented in Table 17.  

**Table 18:** Multinomial logistic regression for willingness to engage in community healthcare (reference = “I will not“) Bold font indicates statistical significance. RQWL: Regional quota without loan, RQP: Regional quota program

Willingness	Variable	Statistic	p-value	Adjusted OR (95% CI)
"Not decided"	Grade (1–6)	z=-1.28	0.201	0.93 (0.82-1.04)
	Gender: Female (ref: male)	z=-0.662	0.508	0.86 '0.55-1.349
	RQPWL	z=2.16	0.031	5.33 (1.17-24.3)
	RQP	z=0.923	0.356	1.91 (0.48-7.53)
	Extraversion	z=-1.53	0.127	0.96 '0.90-1.01)
	Openness to experience	z=-0.435	0.663	0.99 (0.94-1.04)
	Agreeableness	z=1.81	0.0705	1.05 (0.996-1.11)
	Conscientiousness	z=-0.120	0.905	1.00 (0.96-1.04)
	Neuroticism	z=0.125	0.901	1.00 (0.953-1.06)
"I will"	Grade (1–6)	z=-0.471	0.637	0.97 (0.85-1.11)
	Gender: Female (ref: male)	z=1.48	0.138	1.44 (0.89-2.34)
	RQPWL	z=3.56	<0.001	14.7 (3.34-64.6)
	RQP	z=5.66	<0.001	31.0 (9.43-102)
	Extraversion	z=0.982	0.0128	0.92 (0.86-0.98)
	Openness to experience	z=1.69	0.092	1.05 (0.99-1.11)
	Agreeableness	z=2.56	0.011	1.08 (1.02-1.15)
	Conscientiousness	z=-0.107	0.915	1.00 (0.95-1.04)
	Neuroticism	z=0.665	0.506	1.02 (0.96-1.08)
